# Detection and prioritization of COVID-19 infected patients from CXR images: Analysis of AI-assisted diagnosis in clinical settings

**DOI:** 10.1016/j.csbj.2024.11.045

**Published:** 2024-12-05

**Authors:** Carlo Alberto Barbano, Luca Berton, Riccardo Renzulli, Davide Tricarico, Osvaldo Rampado, Domenico Basile, Marco Busso, Marco Grosso, Marco Grangetto

**Affiliations:** a Computer Science Dept., University of Turin, Italy; b Azienda Sanitaria Locale TO3, Italy; c AITEM Solutions s.r.l., Italy; d Medical Physics Department, A.O.U. Città della Salute e della Scienza di Torino, Turin, Italy

## Abstract

In this paper, we present the significant results from the Covid Radiographic imaging System based on AI (Co.R.S.A.) project, which took place in Italy. This project aims to develop a state-of-the-art AI-based system for diagnosing Covid-19 pneumonia from Chest X-ray (CXR) images. The contributions of this work are manifold: the release of the public CORDA dataset, a deep learning pipeline for Covid-19 detection and prioritization, the clinical validation of the developed solution by expert radiologists, and an in-depth analysis of possible biases embedded in the data and in the models, in order to build more trust in our AI-based pipeline. The proposed detection model is based on a two-step approach that provides reliable results based on objective radiological findings. Our prioritization scheme ensures the ordering of the patients so that severe cases are presented first. We showcase the impact of our pipeline on radiologists' workflow with a clinical study, allowing us to assess the real benefits in terms of accuracy and time efficiency. Project homepage: https://corsa.di.unito.it/.

## Highlights

• Presentation of the Co.R.S.A. research project, from the data collection (publicly available) to the clinical validation.
• Deep learning pipeline for COVID-19 detection.
• Deep learning pipeline for COVID-19 prioritization.
• Impact of AI-assisted tools in clinical settings for COVID-19.
• In-depth analysis of possible biases embedded in the data and in the models.

## Graphical abstract

**Figure:**
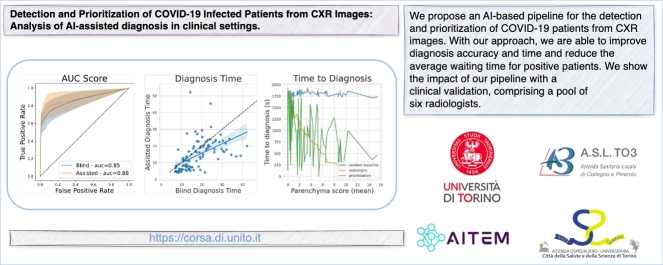

## 1 Introduction

COVID-19 is an infection of the respiratory system that mainly affects the lungs. Common symptoms include fever, fatigue, cough, breathing difficulties, loss of smell, and loss of taste. COVID-19 is very contagious and, starting from the beginning of 2020, quickly spread worldwide, resulting in the COVID-19 pandemic [1]. By August 2024, the global tally surpassed 770 million confirmed cases, resulting in over 7 million deaths.^1^ Radiological imaging has since become recognized as a valuable tool in detecting COVID-19, even at its early stages. Both chest X-rays (CXR) and computed tomography (CT) scans play a significant role in diagnosis. Clinical studies indicate that CT scans are the most accurate imaging modality for diagnosing COVID-19 [2]. However, logistical challenges such as the need for sanitizing rooms, ensuring proper protective gear, radioprotection, and the high costs associated with CT make its routine use difficult in early patient management. In contrast, CXR is often the preferred method in emergency settings due to its ease of use, lower radiation exposure, and comparatively lower costs.

In 2022, the Piedmont Region financed the Covid Radiographic Imaging System based on AI (Co.R.S.A.) initiative. This collaborative effort between the University of Turin, two hospital radiology units (A.O.U Cittá della Salute e della Scienza, Azienda Sanitaria ASL TO3), and the company REGOLA aimed to create and field-validate an advanced AI system to assist in diagnosing COVID-19 pneumonia from CXR images.

The main achievements and contributions of this work include:

1. The creation and public release of the CORDA dataset, along with a thorough assessment of the potential stratification factors (e.g., location, gender, imaging techniques) that could affect AI tools;
2. The development of a robust deep learning framework for prioritizing and detecting COVID-19 cases;
3. Clinical validation of the system by a panel of expert radiologists.

Our validation protocol stands out as the most significant among these contributions, and it also includes the evaluation of possible biases in the CORDA dataset. Prior to integrating an AI tool into clinical practice for diagnostic purposes, it must undergo a thorough evaluation to uncover any limitations or potential biases in its classification outcomes. We consider this analysis essential for establishing trust in these systems and minimizing the likelihood of errors in their application. By incorporating expert assessment, this project bridges that gap, greatly enhancing the clinical relevance of its findings. Understanding the limitations of AI tools and potential biases of data is especially relevant in clinical settings. The importance of such a topic is also noted by [3], which highlights how discovering these limitations enables managers and medical personnel to better understand the risks and benefits of each tool. If not addressed, opacity in the behavior of AI tools may lead to more uncertainty in professionals [4]. For this reason, it is important to design user-centered systems which are based on human-in-the-loop, especially when dealing with AI tools [5].

Although the peak of the COVID-19 pandemic has subsided, the outcomes of this project lay a solid foundation for the quick deployment of similar diagnostic systems in future epidemic responses, ensuring preparedness for emerging public health threats.

## 2 Results

In this section, we present our findings on the impact of our AI-assisted pipeline in clinical settings. We also perform an in-depth analysis of potential biases in the data. First, in Sec. 2.1, we focus on COVID-19 detection; then, in Sec. 2.2, we analyze potential sources of biases either in the data or in the models; lastly, in Sec. 2.3 we present the results of our prioritization pipeline.

### 2.1 Impact of COVID-19 CXR screening

This section focuses on assessing the impact of AI-aided diagnosis in the radiologists' workflow, one of the key aspects of our project. To this end, the clinical validation focuses on measuring two Key Performance Indicators (KPIs): *i)* the accuracy of the radiologists' diagnosis *ii.)* the time needed to formulate it.

#### 2.1.1 Materials and tools

##### Training data

For training the proposed deep learning models, we employ the CORDA dataset [6], comprising 1601 CXR images of both COVID-19 positive and negative patients. We made the dataset publicly available for download in January 2023 [6]. This dataset aims to provide a multi-center collection of radiographic images for COVID-19 detection, in order to build more robust machine learning algorithms and models. The dataset curation is part of the ongoing project Co.R.S.A. CORDA comprises four different Italian hospitals:

1. A.O.U. Città della Salute e delle Scienza (Molinette), Torino (CDSS);
2. A.O.U. San Luigi Gonzata, Orbassano (SLG);
3. A.O. Mauriziano, Torino (MRZ);
4. Centro Cardiologico Monzino, Milano (MNZ).

The dataset composition is summarized in Table 1. We report the composition of the data in terms of imaging modality (either Computed radiography - CR or Digital radiography - DR) and patients' sex. The ground truth in the dataset for COVID-19 is provided as a binary label (positive or negative). It was obtained by using nasopharyngeal swabs, the gold standard for COVID-19 testing.

**Table 1:** CORDA dataset composition: Covid-19 positive and negative cases, imaging modality and patient sex and age divided by institution.

	COVID		Modality		Sex			Age		
Institution	Neg.	Pos.	CR	DR	M	F	N/A	Min.	Mean/Median	Max.
CDSS	183	362	400	145	331	193	21	16	60.66/61	100
SLG	477	250	97	630	1	1	725	–	–	–
MRZ	35	138	12	161	115	58		22	63.52/65	95
MNZ	0	156	0	156	105	51		28	73.49/72	94

Total	695	906	509	1092	552	303	746	16	70.7/69	100

**External Validation data** As validation data, 100 external images were collected at ASL TO3, 50 of which are from COVID-19 patients and 50 are control cases. These images are not part of the CORDA dataset and were not used for previous phases. This external validation also allows us to assess whether the proposed method can generalize to novel data and sites.

**Fig. 1:**
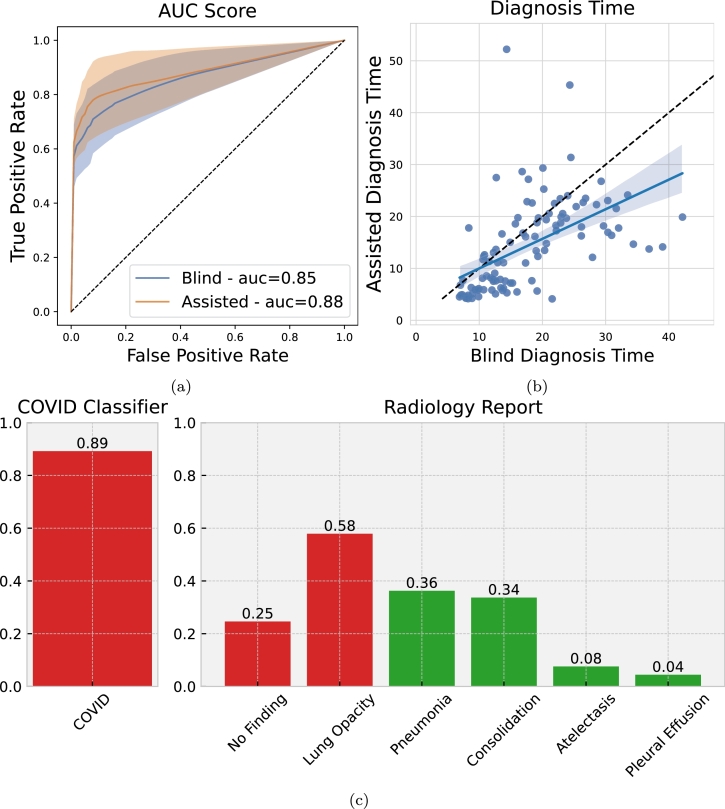
AI-assisted diagnosis helps radiologists make more accurate and faster diagnoses. In **(a)**, we report the average ROC curve of a pool of radiologists during external validation in a blind setting (no AI) and in an AI-assisted setting. In **(b)**, we compare the average diagnosis time (per image) in a blind vs. assisted setting. We notice that the average AUC increases in the AI-assisted setting while the diagnosis is quicker on average. **(c)** Example of AI report in our custom DICOM viewer. This report is shown to the evaluating radiologist alongside the CXR image. The information includes the predicted probability of COVID-19 infection and other relevant lung pathologies. For easiness of readability, probabilities >0.5 are marked in red (except for the “No Finding” class).

**Dicom Viewer** For this validation, we developed a custom DICOM image viewer, which allows deep neural network (DNN) predictions to be shown alongside the analyzed image. Our software automatically collects the diagnosis made by the radiologist for each image and the time taken to make it. Primary functionalities of a standard DICOM viewer are included, such as image manipulation (e.g. zoom, translation) and adjustable windowing (VOI LUT). Fig. 1c depicts an example of DNN predictions as shown to the evaluating radiologist in our viewer. Our DICOM viewer also collects other information such as the time to diagnose each patient.

**Neural Network Training** We employ a two-step approach to train our deep neural networks (DNNs), presented in-depth in [7], [8]. In short, we first train a DenseNet-121 [9] based model on the large-scale dataset CheXpert [10]. CheXpert provides labels for 14 different radiological findings, namely *No Finding, Enlarged Cardiomediastinum, Cardiomegaly, Lung Lesion, Lung Opacity, Edema, Consolidation, Pneumonia, Atelectasis, Pneumothorax, Pleural Effusion, Pleural Other, Fracture, Support Devices.* Additional details about the pre-training protocol are presented in the supplementary material. After pre-training on CheXpert, we perform a transfer learning step on the CORDA dataset [6], employing debiasing regularization [19].

#### 2.1.2 Validation protocol

To perform the clinical evaluation, we employ a pool of 6 radiologists from Ospedale di Rivoli and ASL TO3, with different experience levels. They are summarized in Table 2. For each image, a severity score [11] is assigned by each radiologist upon visual inspection, ranging from 0 (healthy) to 18 (maximum severity). During diagnosis, the radiologists do not know the correct label for a given patient. The experiment is repeated in two settings, with a wash-out period in between:

• *blind* setting: radiologists can only leverage the image to make a diagnosis.
• *AI-assisted* setting: the predicted probabilities of our DNNs are shown alongside the image.

**Table 2:** List of the radiologists involved in the clinical validation study.

Initials and ID	Affiliation	Years of Experience
S. N. (1)	ASL TO3 Rivoli	1
V. N. (2)	ASL TO3 Rivoli	1
D. C. (3)	ASL TO3 Rivoli	2
M. B. (4)	ASL TO3 Rivoli	5
F. L. (5)	ASL TO3 Rivoli	10
P. M. (6)	ASL TO3 Rivoli	15

#### 2.1.3 Validation results

**Fig. 2:**
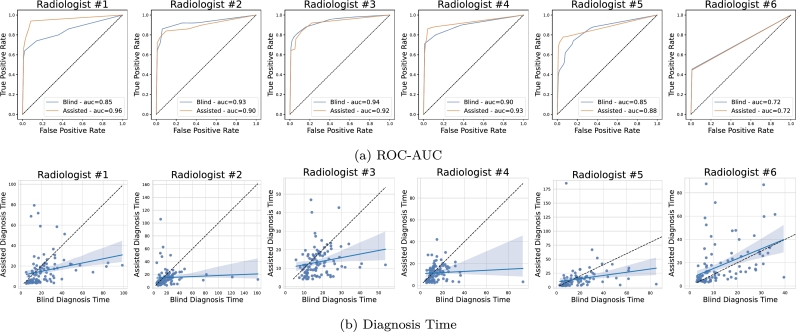
Break-out of diagnosis performance by radiologist. (a) shows the AUC score of blind and AI-assisted evaluation. While for some radiologists, especially those with higher blind scores, we notice a slight decrease in AUC, the improvement is consistent in those who achieve a lower base score. On average, the AUC increases from 0.85 to 0.88, as shown in Fig. 1. (b) compares the blind diagnosis time with the AI-assisted diagnosis time. The blue line represents a linear regression line, and the dashed black line represents the identity. We can see that, on average, the diagnosis time decreases as the fitted line lies below the identity for almost all radiologists.

Fig. 1 shows the overall results of the validation in terms of prediction AUC and diagnosis time. In Fig. 1a, the AUC is computed on the radiologists' average severity score assigned for each patient. We observe an improvement in the assisted setting compared to the blind evaluation. In Fig. 1b, we plot the diagnosis time in the two settings: on average (blue line), the time required for diagnosis decreases in the assisted setting. In Fig. 2, we show a breakout of the results for each participating radiologist for prediction AUC and diagnosis time. In most cases, we observe an improved performance in the assisted setting, with some significant improvement (e.g. AUC increases from 0.85 to 0.96 for radiologist #1). These results show that more accurate and faster diagnosis can be achieved by employing AI systems in a real-world clinical setting.

### 2.2 Evaluation of possible biases in the CORDA dataset

In this section, we evaluate possible sources of bias in the training dataset which can influence the final model. Before using an AI tool for diagnosis in the clinical routine, it should be rigorously evaluated to identify its limitations and possible biases in the classification output. We believe this analysis is fundamental to building trust in such tools and reducing the risk of errors in their usage. We focus our analysis on the differences in the distribution of imaging modality (CR or DR), the number of COVID cases, and patients' sex and age in the different institutions. We also evaluate the differences in the distributions of exposure parameters that influence the image quality of the CXR between positive and negative cases. Furthermore, we analyze whether our model shows significant performance differences among the centers, image modalities, patient sex, and projection type.

#### 2.2.1 Dataset imbalance

As shown in Table 1, the dataset is imbalanced among the different institutions concerning the number of positive and negative cases, imaging modality, and patient sex. The number of images from the different institutions varies widely, mostly from CDSS and SLG. It should be noted that for some patients from CDSS and most of those from SLG, the information regarding the patient's sex (and age) was not available in the DICOM tags, and the sex was labeled as NA. There is also a significant difference in the number of CR and DR images among the different institutions since they have an evident prevalence of one modality over the other; for example, CDSS provides most CR images. Furthermore, the DR images are twice as many as CR images. Moreover, there is an imbalance in the number of positive and negative cases among the different institutions if divided by imaging modality, as seen in Table 3.

**Table 3:** Number of positive and negative cases divided by institution and imaging modality.

Institution	COVID-19	DR Images	CR Images
CDSS	0	16	167
	1	129	233

SLG	0	419	58
	1	211	39

MRZ	0	32	4
	1	129	8

MNZ	0	0	0
	1	156	0

Total	0	467	229
	1	625	280

#### 2.2.2 Differences of the distributions of acquisition parameters

We use the Mann-Whitney U-test, also known as the Wilcoxon test, to assess if there are differences between positive and negative images in the distributions of parameters that may influence image quality. Such differences between the two groups may play a role in the final classification performed by our model. They could introduce a source of bias when the model is applied to external data. The Mann-Whitney U-test is a non-parametric statistical test to compare two distributions and has the advantage of not assuming a specific distribution [12] [13].

**Fig. 3:**
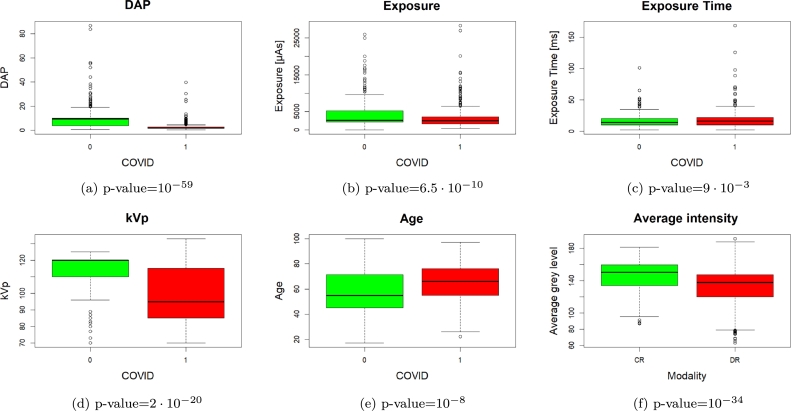
Distributions of the exposure parameters, age, and average image intensity, with the p-value of the Mann-Whitney U-test of each comparison.

The parameters' distributions are shown in Fig. 3. We notice that the dose-area product (DAP) is significantly higher for negative patients (p-value=$10^{-59}$), the exposure (in *μA*) is higher for negative ones (p-value=$6.5\cdot 10^{-10}$). We also noted a significant difference in the distributions of the exposure time (p-value=$9\cdot 10^{-3}$). These differences might be caused by the fact that the CR images of the dataset, which have a higher fraction of negative patients than DR images, usually have higher exposure parameters.

Finally, the value of kVp (which influences the contrast of the image) is significantly lower for the positive cases (p-value=$2\cdot 10^{-20}$). Moreover, we found a significant difference in the average image intensity between CR and DR images, with DR images showing lower values (p-value=$10^{-34}$). This may reflect the intrinsic physical differences between the two imaging modalities. We also compared the age distribution of positive and negative patients, finding that positive patients are significantly older (p-value=$10^{-8}$). This reflects the fact that hospitalization due to COVID-19 is more frequent for older people.

#### 2.2.3 Differences in classification performance

Our bias analysis ends with focusing on the model's performance on different significant subgroups of the dataset to highlight if they influence the classification. We perform this analysis by calculating the AUC of our model for each subgroup, with a 95% confidence interval (C.I.). The confidence intervals are evaluated using the pROC package for R [14], which uses the DeLong statistics to calculate them [15]. The results are presented in Table 4, Table 5.

**Table 4:** Model performance by institution and image modality.

**Table:** Institution AUC (95% C.I.) MRZ 0.82 (0.75-0.89) CDSS 0.75 (0.71-0.79) SLG 0.89 (0.87-0.92) MNZ NA	**Table:** Modality AUC (95% C.I.) CR 0,72 (0,68-0,77) DR 0,91 (0,90-0,93)

**Table 5:** Model performance by patient's sex and projection type.

**Table:** Sex AUC (95% C.I.) M 0,79 (0,75-0,84) F 0,74 (0,69-0,80) NA 0,89 (0,86-0,92)	**Table:** Projection AUC (95% C.I.) AP 0,88 (0,84-0,92) PA 0,86 (0,80-0,92) LAT 0,90 (0,83-0,96)

First, we separate the data by institution. For MNZ, it is impossible to compute an AUC score since all images are positive cases. Our analysis shows that the model has different classification capabilities for different institutions: SLG has the highest AUC, while CDSS has the lowest.

Then, we analyze the data by imaging modality, and we find a significant difference in the classification performance: for CR images, the model achieves an AUC=0.72 (95% C.I.=0.68-0.77), while for DR images, it achieves an AUC=0.91 (95% C.I.=0.90-0.93). This may be due to the different number of images of the two modalities. Still, the intrinsic differences in image quality between CR and DR images may play an essential role in this difference, with DR images having higher contrast and resolution. This may also partially explain the difference in performance among the different institutions, considering the different distribution of CR and DR images: CDSS has the highest fraction of CR images, while SLG has the most DR images.

We also observe that there is a difference in the classification capability considering the sex of the patients: female and male patients have a lower AUC compared to patients with no data regarding their sex, with respectively females with an AUC=0.74 (95% C.I.=0.69-0.80), males with AUC=0.79 (95% C.I.=0.75-0.84) and NA with AUC=0.89 (95% C.I.=0.86-0.92). This may be partially explained by the fact that most NA patients are from institution SLG, which is the one where our model achieves the best classification accuracy. Lastly, we divided the images by the type of projection, according to the DICOM tag “view position”: anterior-posterior (AP), posterior-anterior (PA), or lateral (LAT). The performance of the model does not show a significant difference among the different projections: AP images have AUC=0.88 (95% C.I.=0.84-0.92), PA images have AUC=0.86 (95% C.I.=0.80-0.92) and LAT images have AUC=0.90 (95% C.I.=0.83-0.96).

#### 2.2.4 Takeaways

Our model exhibits different results in different population groups, even though some precautions, such as site regularization, were employed. This is an essential factor to consider when deploying such systems in real-world scenarios, as these differences can ultimately influence the model's predictions. Even with these issues, however, our system can still significantly improve the quality and speed of the diagnosis, as shown by our clinical validation.

### 2.3 Results of the prioritization system

This section presents the results of our AI-based prioritization model for COVID-19 patients. In the context of an emergency department, implementing a tool capable of prioritizing the most suspicious CXRs would be of significant value. Such a tool could facilitate the rapid identification of potential COVID-19 cases and minimize exposure risks in waiting areas. To address this need, we evaluated the effectiveness of a CNN-based prioritization model designed to identify potentially suspicious CXRs and position them at the top of the radiologists' workflow. To evaluate the system's efficiency in prioritizing cases with a higher likelihood of being positive, we analyzed the rankings generated using the Mean Average Precision (mAP) metric [16]. A higher mAP score indicates a greater prioritization of positive cases over negative ones. In the *blind* setting, the mAP was 47%, whereas in the *AI-assisted* setting, we achieved an mAP of 78%. These results demonstrate the effectiveness of the proposed system in assigning higher priority to positive cases compared to negative ones.

**Fig. 4:**
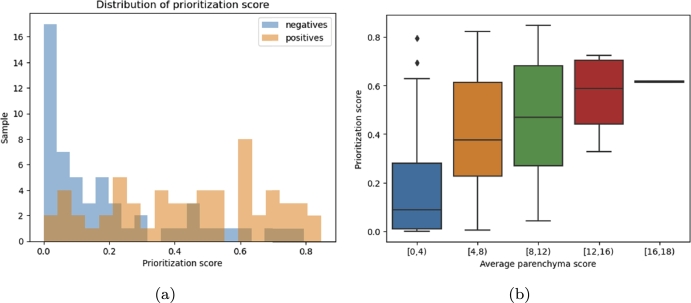
(a) Distribution of prioritization index in the *AI-assisted* setting. (b) Distribution of priority index for different ranges of severity scores.

A more in-depth analysis was conducted by examining the distribution of the prioritization index between positive and negative cases. This distribution is represented in Fig. 4a, highlighting that negative cases are assigned a significantly lower priority than positive ones. Specifically, most non-COVID-19 patients had a prioritization value close to or equal to zero. The median prioritization for negative patients was 11%, while for positive patients it reached 46%, resulting in a substantial gap. Over 60% of negative cases exhibited a prioritization index below 20%, in contrast to only 20% of positive cases. A comparison between the physicians' evaluation in terms of severity score and the prioritization index indicates a general increase in the latter for more severe infection cases, with a correlation of 54%. Fig. 4b demonstrates how the distribution of prioritization values shifts towards higher levels as case severity increases, highlighting a marked prioritization of more severe infections.

To further illustrate the impact of our prioritization pipeline, we conduct a Monte Carlo simulation where each patient's waiting time (time to diagnosis) is computed as the sum of the total time radiologists take to produce a diagnosis for all the previous patients. During the external validation, we tracked the time taken by each radiologist to decide on each patient with our custom DICOM viewer (see Sec. 3.1). For each patient, we compute the normal distribution statistics using the six different measures of diagnosis time (one for each radiologist), and we use them to sample N=10 values. We then employ three different ordering schemes:

1. A random order, where each patient can randomly appear after any other patient.
2. A *oracle* order, where patients are ordered based on the average severity score assigned by radiologists.
3. An order based on the prioritization score of our neural network.

**Fig. 5:**
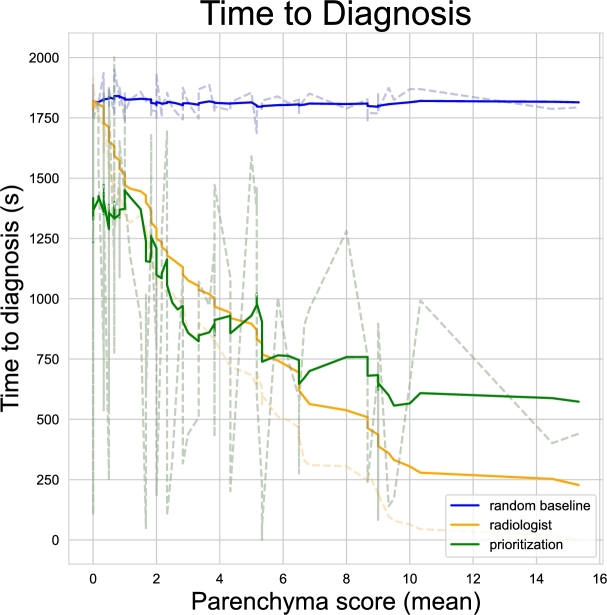
Average waiting time for patients with different severity scores assigned by radiologists with different settings: *blind*, radiologist based and *AI-assisted*.

For each scheme, we repeat the simulation using all the possible values sampled before to obtain an average prioritization index, that we assign to each patient. Intuitively, if the ordering computed by the neural network is close to the ordering obtained by the real scores by radiologists, then our pipeline will successfully prioritize severe patients. The results are illustrated in Fig. 5. The advantage in waiting times for positive patients, between the *prioritization* and *random baseline* settings, is evident, as the waiting time tends to decrease with higher parenchyma scores. In contrast, the random baseline remains nearly constant. Furthermore, looking at the *prioritization* and *radiologist* curves, we can clearly observe how they follow the same trend, meaning that the prioritization is actually working as intended. Notably, for the most severe cases, we can observe a reduction of waiting times of a factor of almost x10.

## 3 Methods

In this section we present the methods that we employed for training our neural models.

### 3.1 COVID-19 detection

The method that we propose consists of two steps: first, we pre-train a deep neural network on a large-scale CXR dataset, with the aim of detecting objective radiological findings, then we apply transfer learning to train a Covid-19 classifier on the CORDA dataset. As previously shown in [8], this approach provides reliable results. Additionally, as shown in [7], we also employ the FairKL regularization technique [7] to partially mitigate the site effect of medical data [17].

**Pre-training on objective radiological findings** We leverage a large-scale dataset, *CheXpert* [10], which contains annotation for different kinds of common radiological findings that can be observed in CXR images (like opacity, pleural effusion, cardiomegaly, etc.). This large dataset is well suited for multi-label classification tasks; in fact, more than one finding can be commonly observed simultaneously in ill patients' lungs. CheXpert provides 14 different types of observations for each image in the dataset. We use the setup described in [8], consisting of a slightly modified DenseNet-121 [9] architecture. More details about pre-training can be found in the Appendix.

**Covid-19 Prediction** To obtain a COVID-19 classifier, we employ the DNN encoder pre-trained on CheXpert as a frozen feature extractor on the CORDA dataset. We train a fully connected binary classifier for the final prediction, using the standard binary cross-entropy loss (BCE). More details about pre-training can be found in the Appendix.

### 3.2 Prioritization

**Fig. 6:**
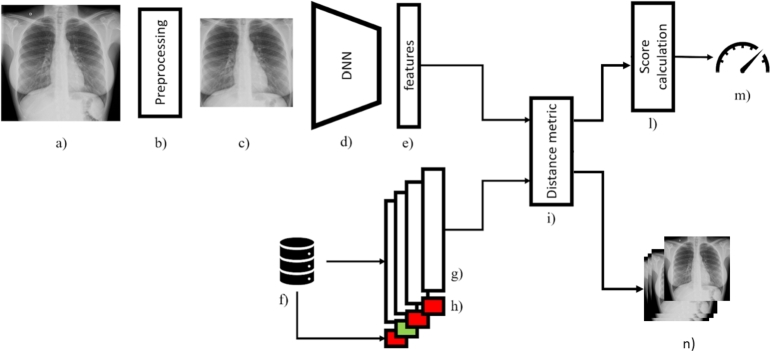
The prioritization system architecture. The image (a) is pre-processed by algorithm (b) into the new image (c) which is elaborated by the deep neural network (d) to extract features (e). Computer features are compared by the distance metric (i) with the previously extracted features (g) stored in the database (f) with their labels (h). The features (g) have been calculated on past known cases. Using the similarity information elaborated by (i) and labels (h) a COVID-19 score (m) is computed by stage (l). The set of most similar cases from the past (n), used for the computation of score (m), is returned to support doctor's diagnosis and to provide an interpretation of the result.

The prioritization system is built upon a deep neural network based on the *DenseNet-121* architecture [9]. This network leverages metric learning to assess the similarity between images from the CORDA dataset [6]. The weights for the deep neural network were initialized using those obtained from training the same architecture on the *ImageNet* classification task. No fine-tuning was applied, ensuring that the performance of the CNN was evaluated using the pre-trained *ImageNet* weights. The structure of the system is depicted in Fig. 6 and is organized as follows:

**Preprocessing**: after resize, U-Net lung segmentation model [18] crops down CXRs producing a final image that just includes the lungs, excluding parts of the image that can add details unrelated to Covid-19. Picture pixel's intensity is finally normalized by subtracting its average and dividing by its standard deviation.

**Feature extraction**: preprocessed images are projected into the target feature space using the deep convolutional neural network *DenseNet-121*. In this case, the original architecture has been truncated before the output classification layer, resulting in a convolutional feature extractor that yields a 1024-dimensional projection of the image provided as input. Consider a pre-processed picture *x* from the set of all possible pictures *X*, and a deep neural network model M(⋅), we have:

(1) $$\forall x\in X\to M(x)=z\in F\subseteq R^{1024},$$

where *z* is the feature vector corresponding to the image *x* and belongs to the target feature space F. With the notation $x_{i}$ the *i*-th sample in *X* and $z_{i}=M(x_{i})$, consider $y_{i}\in S\subseteq R$, the label associated with $x_{i}$, the goal is to design M(⋅) such that:

(2) $$\forall i,j,k\in N:\{||y_{i},y_{j}||<||y_{i},y_{k}||\}\to \{||z_{i},z_{j}||<||z_{i},z_{k}||\}$$

where i,j,k indicate three samples in set *X* and ||a,b|| is the distance between points *a* and *b*. The meaning of Eq. (2) is that if the distance between $y_{i}$ and $y_{j}$ is less than the distance between $y_{i}$, $y_{k}$, then the distance between the embedding $z_{i}$ and $z_{j}$ should be less than the distance between $z_{i}$ and $z_{k}$. This distance metric can be chosen conveniently to maximize the performance, using an iterative process, among the ones presented in the literature.

**Distance-based regression**: To compute the final prediction, the method exploits the distance in the target feature space between the query image and the reference pictures for which the label is known. Consider a set $X^{ref}$ of $n^{ref}$ reference pictures from the CORDA dataset with relative label $Y^{ref}$, such that given a $x_{i}^{ref}\in X^{ref}$ its label is $y_{i}^{ref}$, $\forall i=[1,2,...n_{ref}]$. The variable $y_{i}^{ref}$ takes value 1 if picture $x_{i}^{ref}$ is related to Covid-19 positive patient, 0 otherwise. The methodology computes *feature reference set* $Z^{ref}$ as:

(3) $$Z^{ref}:=\{M(x_{i}^{ref})\;\forall i=[1,2,...n_{ref}]\}$$

Whenever it is requested to predict the prioritization score for a query picture $x^{q}$, its projection in the target feature space is computed as:

(4) $$z^{q}=M(x^{q})$$

The distance between the resulting 1024-dimensional vector $z^{q}$ and the set of reference vectors $Z^{ref}$ is then computed. The elements in the feature reference set are sorted from the nearest to the most distant and related elements in $Y^{ref}$ are stored in the sorted list $Y^{ref,sort}$. To compute the final prediction $\overset{ˆ}{y^{q}}$, the proposed algorithm computes:

(5) $$\overset{ˆ}{y^{q}}=\frac{1}{\sum_{i=1}^{m}w_{i}}\sum_{i=1}^{m}w_{i}\cdot y_{i}^{ref,sort},\;y_{i}^{ref,sort}\in Y^{ref,sort}\;\forall i=1,2,...n^{ref}$$

where $m\leq n^{ref}$ is a parameter of the algorithm, regulating the number of nearest neighbors considered in the score estimation, and $w_{i}$ is a weight coefficient used to regulate the contribution of each sample. To tune the first, an iterative process has to be put in place: for increasing values of *m*, the performance of the method is evaluated, looking for the best trade-off. In our case, we tuned *m* on the CORDA dataset, using a cross-validation approach. The best results were achieved with m=10. The definition of the weight coefficients can be performed empirically, depending on their distance in the target feature space or their position in the ordered list $Y^{ref,sort}$. The coefficients $w_{i}$ can be used to incorporate distance into the final score computation. The idea is that closer samples should have a greater weight in the average computation, while less similar samples should weigh less. We experimented with different formulations and we found that a logarithmic decay of $w_{i}$ produced the most favorable outcome:

(6) $$w_{i}=\left(\frac{1}{\log(i+1)}-\alpha \right)\beta ,\;\alpha =\frac{1}{log(N+1)},\;\beta =\frac{1}{1-\alpha }$$

where *N* is the number of samples. Note that the reference set $Z^{ref},Y^{ref}$ contains both positive and negative samples: this is needed because, given a query image $x^{q}$ we aim to determine whether $x^{q}$ is positive or negative, by computing the similarity with both classes in the reference set. The higher ${\overset{ˆ}{y}}^{q}$ will be, the more priority we will assign to $x^{q}$.

**Diagnostic workflow prioritization**: The described process is applied to each CXR in the diagnostic queue, assigning a prioritization score for each case. The workflow is then reorganized by arranging the cases in descending order of priority, from highest to lowest.

## 4 Conclusions

In this work, we have shared the outcomes of the Co.R.S.A. project, which focused on *i)* creating an open-access dataset for Covid-19 diagnosis using CXR images, along with a thorough assessment of the potential biases *ii)* developing a robust deep learning pipeline for automatic classification and prioritization, and *iii)* conducting clinical validation in a real-world setting with expert radiologists. This project encapsulates our extensive efforts in COVID-19 detection over the past few years. Although the most critical phase of the pandemic has passed, the groundwork laid by this initiative provides a strong foundation for rapidly responding to future epidemics, supported by the collaborations established with key hospitals and radiology units.

Finally, we addressed some potential limitations of the dataset used to train our model. Identifying and accounting for such limitations is crucial before any AI tool can be applied in a clinical setting, particularly given the challenges of acquiring a diverse and balanced collection of clinical images.

## Compliance with ethical standards

This study was performed in line with the principles of the Declaration of Helsinki. Approval was granted by Comitato Etico Interaziendale A.O.U. San Luigi di Orbassano, AA.SS.LL. TO3, TO4, TO5 (10/24/2022/ No. 153/2022).

## CRediT authorship contribution statement

**Carlo Alberto Barbano:** Writing – review & editing, Writing – original draft, Software, Methodology, Data curation, Conceptualization. **Luca Berton:** Writing – original draft, Visualization, Validation, Formal analysis. **Riccardo Renzulli:** Writing – review & editing, Writing – original draft, Investigation. **Davide Tricarico:** Writing – original draft, Visualization, Methodology. **Osvaldo Rampado:** Supervision. **Domenico Basile:** Data curation. **Marco Busso:** Supervision, Data curation. **Marco Grosso:** Validation, Supervision, Project administration, Data curation. **Marco Grangetto:** Writing – review & editing, Writing – original draft, Validation, Supervision, Project administration, Methodology, Investigation, Funding acquisition, Conceptualization.

## Declaration of Competing Interest

The authors declare the following financial interests/personal relationships which may be considered as potential competing interests: Marco Grangetto reports financial support was provided by 10.13039/501100009885Piedmont Region. If there are other authors, they declare that they have no known competing financial interests or personal relationships that could have appeared to influence the work reported in this paper.

## Appendix A Supplementary material

MMC Supplementary material for “Detection and Prioritization of COVID-19 Infected Patients from CXR Images: Analysis of AI-assisted diagnosis in clinical settings.” Here we report additional details about the methods and training protocol presented in the main text. MMC

## Footnotes

1 https://covid19.who.int/.

## References

[1] Zu Zi Yue, Jiang Meng Di, Xu Peng Peng, Chen Wen, Ni Qian Qian, Lu Guang Ming (2020). Coronavirus disease 2019 (covid-19): a perspective from China. Radiology.

[2] Shi Heshui, Han Xiaoyu, Jiang Nanchuan, Cao Yukun, Alwalid Osamah, Gu Jin (2020). Radiological findings from 81 patients with covid-19 pneumonia in Wuhan, China: a descriptive study. Lancet Infect Dis.

[3] Lebovitz Sarah, Levina Natalia, Lifshitz-Assaf Hila (2021). Is ai ground truth really true? The dangers of training and evaluating ai tools based on experts' know-what. MIS Q.

[4] Roggema Rob, Chamski Robert (2022). The new urban profession: entering the age of uncertainty. Urban Sci.

[5] Grønsund Tor, Aanestad Margunn (2020). Augmenting the algorithm: emerging human-in-the-loop work configurations. J Strateg Inf Syst.

[6] Alesina Marta, Barbano Carlo Alberto, Berzovini Claudio, Busso Marco, Calandri Marco, De Pascale Agostino (Jan. 2023). CORDA dataset. 10.5281/zenodo.7821611.

[7] Barbano Carlo Alberto, Renzulli Riccardo, Grosso Marco, Basile Domenico, Busso Marco, Grangetto Marco (2024). 2024 IEEE international symposium on biomedical imaging (ISBI).

[8] Barbano Carlo Alberto, Tartaglione Enzo, Berzovini Claudio, Calandri Marco, Grangetto Marco (2022). Image analysis and processing – ICIAP 2022.

[9] Huang G., Liu Z., Van Der Maaten L., Weinberger K.Q. (jul 2017). 2017 IEEE conference on computer vision and pattern recognition (CVPR).

[10] Irvin Jeremy, Rajpurkar Pranav, Ko Michael, Yu Yifan, Ciurea-Ilcus Silviana, Chute Chris (Jul. 2019). Chexpert: a large chest radiograph dataset with uncertainty labels and expert comparison. Proc AAAI Conf Artif Intell.

[11] Borghesi Andrea, Maroldi Roberto (2020). Covid-19 outbreak in Italy: experimental chest x-ray scoring system for quantifying and monitoring disease progression. Radiol Med.

[12] Mann Henry B., Whitney Donald R. (1947). On a test of whether one of two random variables is stochastically larger than the other. Ann Math Stat.

[13] Wilcoxon Frank (1945). Individual comparisons by ranking methods. Biom Bull.

[14] Robin Xavier, Turck Natacha, Hainard Alexandre (2011). Proc: an open-source package for r and s+ to analyze and compare roc curves. BMC Bioinform.

[15] DeLong Elizabeth R., DeLong David M., Clarke-Pearson Daniel L. (1988). Comparing the areas under two or more correlated receiver operating characteristic curves: a nonparametric approach. Biometrics.

[16] Tricarico Davide, Calandri Marco, Barba Matteo, Piatti Clara, Geninatti Carlotta, Basile Domenico (2022). Convolutional neural network-based automatic analysis of chest radiographs for the detection of covid-19 pneumonia: a prioritizing tool in the emergency department, phase i study and preliminary “real life” results. Diagnostics.

[17] Glocker Ben, Robinson Robert, Castro Daniel C., Dou Qi, Konukoglu Ender (2019). Machine learning with multi-site imaging data: an empirical study on the impact of scanner effects. https://arxiv.org/abs/1910.04597.

[18] Ronneberger Olaf, Fischer Philipp, Brox Thomas (2015). Medical image computing and computer-assisted intervention–MICCAI 2015: 18th international conference, Munich, Germany, October 5-9, 2015, proceedings, part III 18.

[19] Barbano Carlo Alberto, Dufumier Benoit, Tartaglione Enzo, Grangetto Marco, Gori Pietro (2023). The eleventh international conference on learning representations.

