# Supplementary material for: Detection and prioritization of COVID-19 infected patients from CXR images: Analysis of AI-assisted diagnosis in clinical settings
Source: Comput Struct Biotechnol J. 2024 Dec 5;24:754–61. doi: 10.1016/j.csbj.2024.11.045 (PMC11681887; doi:10.1016/j.csbj.2024.11.045)
Supplement: MMC — Supplementary material for “Detection and Prioritization of COVID-19 Infected Patients from CXR Images: Analysis of AI-assisted diagnosis in clinical settings.” Here we report additional details about the methods and training protocol presented in the main text. [file mmc1.pdf]

Appendix A. Experimental setup

Appendix A.1. Pre-training on CheXpert

We pre-train a DenseNet-121 [9] with SGD, with a learning rate of 0.01 and a weight decay of 10^{-4}. We train for 10 epochs using 224x224 size and then we upscale to 448x448 for another 10 epochs. We employ a learning rate decay policy based on the plateau of the loss computed on the validation set (30% of the training set), with patience of 5 epochs and a factor of 0.1.

Appendix A.1.1. Loss function

The loss used for training is a weighted binary cross entropy (wBCE):

$$L_n = -w_n \cdot [y_n \log(x_n) + (1 - y_n) \log(1 - x_n)] \tag{A.1}$$

where, for a given sample, y_n is the ground truth label for the n-th class, x_n is the model probability prediction and w_n is the weight associated to the n-th class. The weights are used to address class imbalance and missing labels, with the following scheme:

$$w_n = \begin{cases} 1 + S_n^+/S_n^- & \text{if } y_n = 0 \\ 1 + S_n^-/S_n^+ & \text{if } y_n = 1 \\ 1 & \text{if } y_n = 0.5 \end{cases} \tag{A.2}$$

where S_n^- and S_n^+ respectively represent the cardinality of negative and positive samples for the n-th class. Hence, uncertain samples will have a lower influence during the training process, while being pushed either towards 0 or 1 by the higher weight of certain samples in the same class.

Appendix A.1.2. Model architecture

We employ the Hierarchical Residual (HR) architecture [8], which is composed of a two-layer fully-connected classifier stacked on top of a DenseNet-121 encoder. In the HR architecture, the first layer of the classifier is used to predict the 8 top-level classes of CheXpert (No Finding, Pneumothorax, Support Devices, Pleural Effusion, Pleural Other, Enlarged Cardiomediastinum, Lung Opacity, Fracture), while the second layer is used to classify the 6 bottom-level classes of CheXpert (Edema, Consolidation, Pneumonia, Lesion, Atelectasis, Cardiomegaly) using the output of the encoder of the first classification layer. Additional details can be found in [8].

Appendix A.2. Transfer Learning on CORDA

The experimental setup we follow is described in [7]. We train the final classifier (composed of two fully connected layers) on the frozen DenseNet-121 backbone for 100 epochs, using SGD as an optimizer, with a learning rate of 0.01, decayed with a cosine schedule, a batch size of 32 and an image size of 448x448. We also employ an augmentation scheme composed of random crop, rotation, and cutout. As demonstrated in [7], we employ the FairKL regolarization [19] in order to partially reduce the site effect of images during training.

Appendix A.3. Code for COVID-19 classification

Code for the COVID-19 classification pipeline can be found at `https://github.com/corsa-project/covid-classification/`.

Appendix B. FairKL for Site Regularization

From a debiasing point of view, we define as bias-aligned all those samples that share the same acquisition site w.r.t. to a given *anchor* sample, and bias-conflicting the ones that do not. Also, we define as *positive* the samples that share the same target class as the anchor. We employ the recently proposed FairKL [19] regularization technique, which aims at minimizing the Kullback-Leibler divergence of the distance distributions in the latent space of positive bias-aligned $B_{+,b}$ and positive bias-conflicting and $B_{+,b'}$:

$$\mathcal{R}^{FairKL} = D_{KL}(B_{+,b}||B_{+,b'}) \tag{B.1}$$

This regularization term aims to make samples of the same class indistinguishable in the latent space based on the acquisition site. This choice is motivated by the possible correlation between the target label, modality, and source institution, as shown in Tab. 1. The final objective function we optimize is thus $J = \mathcal{L}^{BCE} + \lambda\mathcal{R}^{FairKL}$, with $\lambda \geq 0$.
